# Divergent phenotypes in siblings with identical novel mutations in the *HNF-1α* gene leading to maturity onset diabetes of the young type 3

**DOI:** 10.1186/s12881-016-0297-z

**Published:** 2016-05-04

**Authors:** Birgit Knebel, Susanne Mack, Jutta Haas, Mona Kathrin Herman-Friede, Simone Lange, Oliver Schubert, Jorg Kotzka, Dirk Muller-Wieland

**Affiliations:** Institute of Clinical Biochemistry and Pathobiochemistry, German Diabetes Center at the Heinrich-Heine-University Duesseldorf, Leibniz Center for Diabetes Research, Duesseldorf, Germany; Institute for Diabetes Research, Department of General Internal Medicine, Asklepios Clinic St. Georg, Asklepios Campus Hamburg, Medical Faculty of Semmelweis University, Hamburg, Germany; Diabetes Care Center Buxtehude, Buxtehude, Germany

**Keywords:** Maturity-onset diabetes of the young, HNF1A-MODY, *HNF-1α*, Metabolic syndrome, Metabolic challenge, Phenotype variety

## Abstract

**Background:**

Maturity onset diabetes of the young (MODY) is an autosomal dominant form of non–insulin-dependent diabetes mellitus caused by mutations in at least 13 different genes. The hepatocyte nuclear factor (*HNF*)-*1α* gene is affected in the most common form (HNF1A-MODY [*MODY3*]).

**Case presentation:**

We describe the co-inheritance of a novel heterozygous missense mutation c.1761C > G (p.Pro588Ala) with a novel complex deletion insertion mutation (c.1765_1766delinsGCCCGfs86*) in the *HNF-1α* gene among affected members of one family. Both mutations were present in the affected patients and neither was present in unaffected family members. The family had not only inheritance of MODY but also increased susceptibility to type 2 diabetes. Therefore one family member had classical type 2 diabetes including metabolic syndrome aggravated by a genetic predisposition in the form of HNF1A-MODY.

**Conclusion:**

The presence of common type 2 diabetes features should not detract from the possibility of MODY in patients with a striking autosomal-dominant family history.

## Background

Maturity onset diabetes of the young (MODY) comprises a group of clinically and genetically heterogeneous familial disorders with a clinical appearance similar to non–insulin-dependent type 2 diabetes that is caused by mutations in at least 13 different genes (*MODY1* to *MODY13*) [1, 2]. MODY is characterized by autosomal dominant inheritance and early onset diabetes, but otherwise the phenotype appears to be heterogeneous [1–3]. HNF1A-MODY (MODY3) is the most common form of the disorder in white populations and is caused by mutations and, rarely, deletions affecting *HNF-1α*, a transcription factor that functions in the transcriptional network necessary for liver as well as pancreatic β-cell development and function [4]. HNF1A-MODY is a clinically progressive phenotype with progressive hyperglycemia, glycosuria, and decreased renal function or microvascular complications, which can be reduced substantially by early treatment [1–3, 5]. We report siblings with novel HNF1A-MODY mutations but with different clinical manifestations. This finding may be due to an additional maternal predisposition for type 2 diabetes, which has aggravated the genetic susceptibility burden for the diabetes phenotype in one of the siblings.

## Case presentation

The index patient (female, age 43 years at examination) and five further family members agreed to undertake molecular analyses. The proband and three family members were analyzed for serum metabolic parameters; two patients (the index female patient and her male sibling) agreed to ultrasound examination, an interventional oral glucose tolerance test (oGTT) and lipid tolerance testing. For ethical reasons family members age <18 years and individuals with further overt clinical symptoms did not undergo interventional investigations. All interventions were performed at the Asklepios Clinik St. Georg (Hamburg, Germany). Written informed consent was obtained from all participants (parents provided informed consent on behalf of subjects age <18 years). The study protocol and procedure were approved by the parents of the participating children and the ethical committee of the University of Hamburg. The study was conducted according to principles laid out in the Declaration of Helsinki.

### Determination of metabolic parameters

Blood and serum samples were collected according to standard protocols. Automated standard systems for measuring clinical variables, including insulin, glucose, hemoglobin A1c, cholesterol (including high-density and low-density lipoprotein) and triglycerides, were used. Standard surrogate indexes were calculated from fasting blood glucose and plasma insulin concentrations as follows: quantitative insulin sensitivity check index QUICKI = 1/(log(I0) + log(G0)), where I0 is fasting insulin (μU/ml) and G0 is fasting glucose (mg/dl); and homeostatic model assessment of insulin resistance HOMA-IR = (G0 * I0)/22.5, with fasting glucose expressed as mmol/l and fasting insulin expressed as μU/ml. Creatinine, albumin and glucose concentrations were determined from urine samples. To exclude the presence of type 1 diabetes or latent autoimmune diabetes of adults, measurement of antibodies against insulin, GAD, thyrosine phosphatase (IA2), and islet cells (ICA) were determined. Blood pressure, ophthalmoscopy and angiography of lower extremities were determined in 3 probands.

### Molecular analyses

Genomic DNA was extracted from whole blood using the blood extraction kit (Qiagen, Hilden, Germany). The targeted resequencing approach of glucokinase, *HNF-1α*, *HNF-1β*, *HNF-4α*, *IPF-1/PDX-1* and *NeuroD1/β2* including all splice variants and related promoter areas corresponding to the molecular basis of the most prevalent MODY1 to MODY6 have been reported [6]. Nucleotide numbering of *HNF-1α* was given according to NM_000545.5.

### Metabolic testing

To determine fat tolerance, a breakfast with a total energy content of 1080 kcal (47 % fat, 40 % carbohydrates and 13 % proteins) was served after a 10-h fasting period under current medication. Blood was drawn before the meal (0 min) and 120 min and 240 min after food consumption [7]. To test oGTT, blood samples were drawn before (0 min) and 60 min and 120 min after a 75 mg oral glucose load under current antidiabetic therapy.

## Results

The index patient reported early onset of diabetes at age 25 years. No type 1 diabetes specific antibody pattern was detected, but proteinuria and recurrent nephrolithiasis were reported. In the index patient, diabetes had been diagnosed 3 years before the first pregnancy and she developed obstetric diabetic complications during all of her four pregnancies, which were treated with insulin. Two pregnancies were successful, but both newborns developed reversible jaundice, macrosomia and hypoglycemia within the first 2 weeks.

**Fig. 1:**
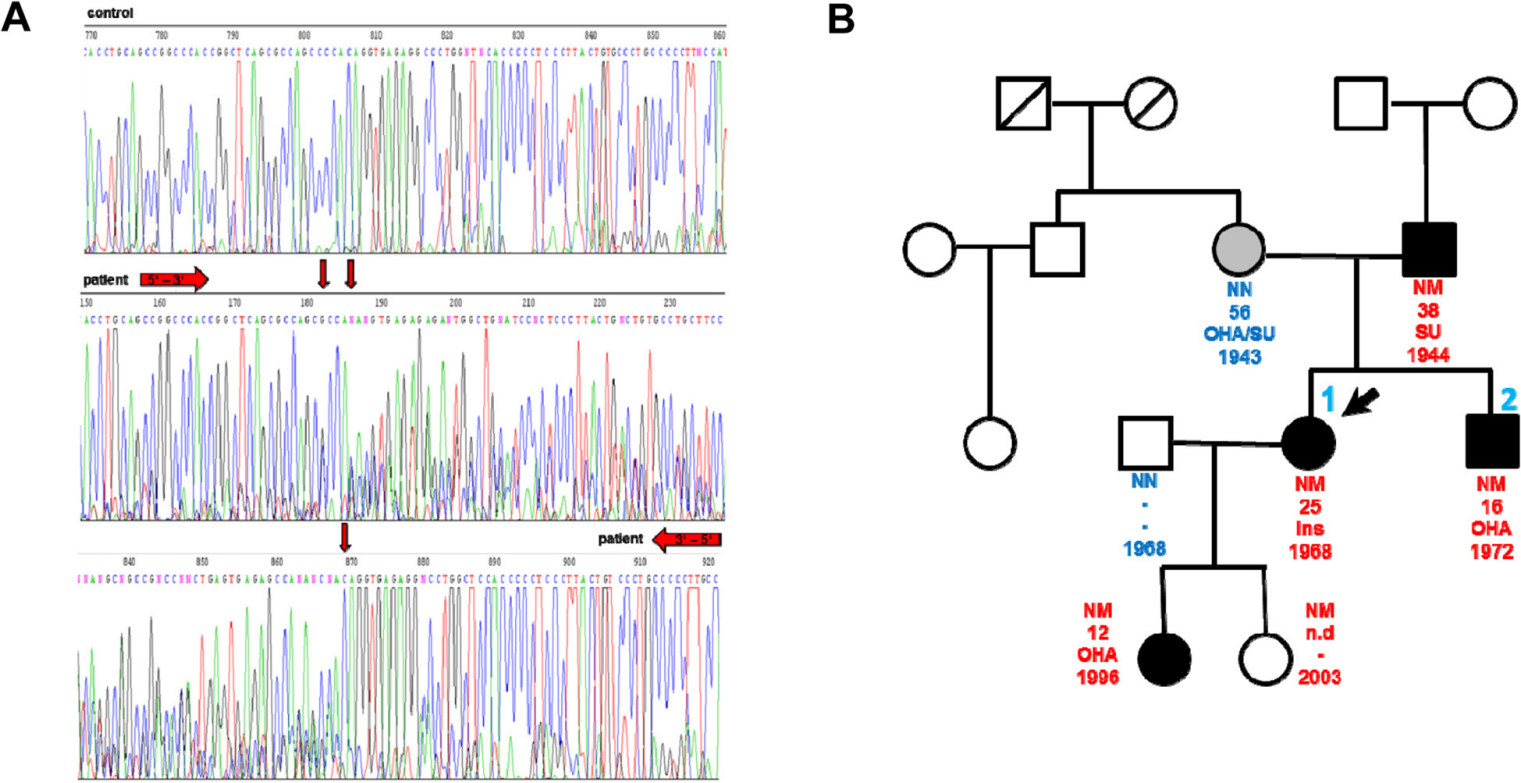
Co-segregation of the mutations and key clinical characteristics. **a** A heterozygous mutation (c.1761C > G (p.Pro588Ala)) and a complex deletion insertion mutation (c.1765_1766delinsGCCCGfs86*) in exon 9 of the *HNF-1α* gene were identified by direct sequencing (upper panel: control, middle patient forward strand, below: patient reverse strand). Horizontal arrows indicate direction of sequencing; vertical arrows indicate sequence alterations. **b** Co-segregation of the *HNF-1α* mutations in the family. The genetic status, age of onset of diabetes, current therapy and date of birth are indicated. Numbers indicate the index patient and the brother. (Black symbol: early onset diabetes phenotype; grey symbol: late onset type 2 diabetes NM: heterozygous mutation present; NN: no mutation; INS: insulin; OHA: oral hypoglycemic agents; SU: sulfonylurea). In detail, at time of investigation the medication was as follows: index patient (insulin, angiotensin-converting-enzyme inhibitors), brother (glinide), daughter (metformin), father (insulin, statin, glibenclamide), and mother (metformin, glibenclamide, statin, angiotensin-converting-enzyme inhibitors)

Examination of the family history revealed that her brother also showed early onset diabetes (at 16 years), whereas her parents were both diagnosed as diabetic in routine health screens. The father was diagnosed at the age of 38 years without microvascular complications, including a stable HbA1c of <7.0 % whereas the mother had typical late onset diabetes diagnosed at age 56 years. Thus the family history indicated type 2 diabetes at the maternal site and diabetes with autosomal heritage and early onset at the paternal site. The positive family history, especially with clinical manifestation at a young age with lack of diabetes-related autoantibodies, is a feature typical of MODY. In accordance with this finding, we identified a novel heterozygous missense mutation c.1761C > G (p.Pro588Ala) in the index patient. Moreover, a novel complex deletion insertion mutation at c.1765_1766delinsGCCCGfs86*, resulting in a frameshift in exon 9 of the *HNF-1α* gene (Fig. 1a), was detected. Both mutations were inherited together and co-segregated with an early onset diabetes phenotype in the family, indicating the genotype–phenotype association over three generations. One child of the family was a carrier of the mutations and diagnosed diabetic at the age of 12 with blood glucose 147 mg/dl and HbA1c 6,9 % (Fig. 1b).

Although the mutations were present in both overweight siblings, their metabolic phenotypes differed (Table 1). The index patient (body mass index 27 kg/m^2^) showed elevated fasting glucose and insulin concentrations, increased HbA1c, moderately elevated triglyceride concentrations and dyslipidemia. Ophthalmoscopy, angiography of the lower extremities and ultrasound examination of the abdomen were performed. No microvascular complications were identified, but low-grade hepatic steatosis and an unaffected pancreas were determined. The brother also showed elevated fasting blood glucose and proteinuria, but no type 1 diabetes-specific antibodies or microvascular complications were detectable. In contrast to the index patient, fasting insulin was normal and HbA1c was significantly increased. Furthermore, features of the metabolic syndrome, including obesity (body mass index 30 kg/m^2^) and dyslipidemia, were more pronounced. Ultrasound examination indicated severe homogenous steatosis and liver enlargement with low-grade splenomegaly and a lipomatous pancreas. The patient was also a tobacco smoker, a further risk factor.

**Table 1:** Clinical biochemical variables in the siblings

Variable	Normal range	Patient 1 (index)	Patient 2
Sex	–	Female	Male
Age at examination, years	–	43	39
Weight (kg)	–	69	93
Height (cm)	–	159	177
Waist circumference (cm)	-	94	108
Body mass index (kg/m^2^)	<25	27	30
Systolic/diastolic blood pressure (mmHg)	120/80	120/80	125/60
Fasting glucose (mmol/l)	4.1–6.1	12.7	12.8
Hemoglobin A1c (%)	4.8–5.9	7.4	8.9
Triglycerides (mmol/l)	<1.7	2.2	6.9
Total cholesterol (mmol/l)	<5.2	7.0	7.3
High-density lipoprotein cholesterol (mmol/l)	>1.0	1.1	0.7
Low-density lipoprotein cholesterol (mmol/l)	<4.2	4.8	3.4
Urea–albumin (mg/l)	<20	22	73
Creatinine (mg/l)	5.3-8.9	3.5	6.2
Albumin/creatinine (g/mol_crea_)	<2.3	4.9	8.2
Glutamic acid decarboxylase-antibodies (mlU/ml)	<70	24.7	<5
Islet cell antibodies (JDF U)	<5	1.3	1.3
Islet antigen 2 antibodies (mlU/ml)	<70	<25	<1.3
C-peptide (nmol/l)	0.3–1.7	0.4	0.7
Insulin (pmol/l)	18.1–173.3	247.2*	76.4
Lipase (mmol*s/l)	<100	46.7	145

Data are shown as mean ± S.D. standard deviation. *: p-values < 0.05 (unpaired *t*-test with the assumption of normal distributed parameters) were accepted as significant

To further characterize potential differences in the metabolic response, the siblings were challenged with oral glucose and separately with a lipid-rich meal (Tables 2 and 3). The index patient showed a diabetes-specific pattern in all variables analyzed in the oGTT. The brother was in the near-normal range except for his triglyceride concentration. As the fasting triglyceride concentration was pathologically high, the values showed only an average decrease to 4.5 mmol/l under glucose load. The lipid profile was aggravated under lipid tolerance tests. Following lipid load, glucose concentrations in the siblings are nearly identical but were elevated compared with the normal range [8–10]. Most strikingly, in the brother, during the lipid tolerance test fasting triglycerides increased from 8.5 mmol/l to >11.4 mmol/l and declined to 10.1 mmol/l within 2 h. Again, the vast increase in triglyceride concentrations in the lipid tolerance test was not observed in the index patient.

**Table 2:** Results from the oral glucose tolerance test in the siblings^a^

	Normal range^b^	Patient 1 (index)	Patient 2
Glucose (mmol/l)			
0 min	<6.1	13.5	11.5
60 min		21.0	21.0
120 min	<7.8	22.2	16.0
180 min		15.8	11.7
Triglyceride (mmol/l)	<1.71		
0 min		3.2	6.1
60 min		3.5	4.9
120 min		3.5	4.4
180 min		3.7	4.2
Total cholesterol (mmol/l)	<5.2		
0 min		6.6	6.5
60 min		6.4	5.7
120 min		6.4	5.7
180 min		6.9	6.0
C-peptide (nmol/l)	0.27–1.72		
0 min		0.4	0.7
60 min		0.8	1.0
120 min		0.6	1.2
180 min		0.7	0.7
Insulin (pmol/l)	18.1–173.6		
0 min		247.2	76.4
60 min		209.7	84.0
120 min		184.0	140.3
180 min		139.3	36.8

^a^Clinical biochemical parameters of the siblings in an oral glucose tolerance test (75 mg oral glucose load) under current medication (patient 1: insulin; angiotensin-converting-enzyme inhibitors; patient 2 glinide; no lipid lowering therapy). ^b^Mean values reported in healthy populations, na: not available

**Table 3:** Results from the lipid tolerance test in the siblings^a^

	Normal value†	Patient 1 (index)	Patient 2
Glucose (mmol/l)			
0 h	5.33	12.3	13.7
2 h	5.0	20.6	20.0
4 h	4.9	15.8	14.2
Insulin (pmol/l)			
0 h	48.6	201.4	48.6
2 h	340.3	305.6	208.4
4 h	132.0	125.0	104.2
Total cholesterol (mmol/l)			
0 h	4.5	5.5	6.8
2 h	4.5	5.6	6.4
4 h	4.5	6.0	6.7
HDL cholesterol (mmol/l)			
0 h	1.3	1.5	0.7
2 h	1.3	1.3	0.8
4 h	1.3	1.3	0.8
Triglycerides (mmol/l)			
0 h	<1.0	2.1	8.5
2 h	<1.6	4.8	11.6
4 h	<1.4	4.3	10.1
U-albumin (mg/l)			
0 h	35-50	30	67
2 h	na	6	46
4 h	na	40	19
Albumin/creatinine (g/mol_crea_)			
0 h	<3.4	5.5	6.2
2 h	na	2.8	4.3
4 h	na	3.4	2.5

HDL, high-density lipoprotein

^a^Clinical biochemical parameters of the siblings in a lipid tolerance test (10-h fasting period followed by breakfast of 1080 kcal [47 % fat, 40 % carbohydrates and 13 % proteins]) under current medication (patient 1: insulin; angiotensin-converting-enzyme inhibitors; patient 2 glinide; no lipid lowering therapy). †Mean values reported in healthy populations, na: not available

## Conclusions

We identified two sequence variations in the *HNF-1α* gene in siblings with divergent clinical manifestations of type 2 diabetes, metabolic syndrome and HNF1A-MODY mutations. One is a heterozygous mutation of c.1761C > G (p.Pro588Ala). The other is a complex deletion insertion mutation at c.1765_1766delinsGCCCGfs86* altering p.Thr589Ser and inserting a further p.P589 + 1 in exon 9 but not affecting the splicing site directly. The mutations identified were not reported in the exome sequencing-based database of human variation consisting of approximately 60,000 individual samples (http://exac.broadinstitute.org/gene/ENSG00000135100). A mutation of 1785C > T (p.Pro588Ser) has been reported in a previous study of MODY families [11], and Colclough et al. [12] observed a frameshift deletion mutation c.1765_1766delCA at the identical site of the mutation identified here. Although both previous reports did not describe the affected families [11, 12], these observations indicate the importance of this area of the *HNF-1α* gene as a mutation hotspot. This, alongside the dominant inheritance and the co-segregation with diabetes phenotype, stresses the hypothesis that the mutation causes the MODY phenotype in the family described here.

*HNF-1α* is composed of three functional domains, and three isoforms are generated by alternative splicing, with different transcriptional properties and tissue expression patterns [13, 14]. Mechanistically both mutations affect the transactivation domain of the *HNF-1α* isoform A with the isoform specific exons 8 to 10 of the transactivation domain, which is mainly expressed in the fetal pancreas [14]. An older age at onset was observed in HNF1A-MODY patients carrying a *HNF-1α* missense mutation affecting specifically the *HNF-1α* (A) isoform (10B) [11]. Interestingly, in vitro studies indicated that truncation of exon 10 decreases transcriptional activation by *HNF-1α* by approximately 50 % [15]. Although the splicing site is not affected directly, as a functional consequence after splicing the reading frame of the entire exon 10 is shifted to generate an 85 AA sequence without any homology to known protein sequences. As the *HNF-1α* isoform A is mainly expressed in the fetal pancreas [14], one may speculate that identified mutations interfere with susceptibility to type 2 diabetes on the level of pancreas functionality or maturation.

MODY in adults is often clinically misdiagnosed as type 1 or type 2 diabetes [3]. Therefore it is tempting to speculate that HNF1A-MODY is under-diagnosed in the ‘sink’ of common type 2 diabetes. HNF1A-MODY is a clinically progressive phenotype with hyperglycemia, glycosuria, and decreased renal function or microvascular complications, and usual associations with microvascular and macrovascular complications commensurate with glycemic control [1, 2, 16]. In our family, HNF1A-MODY presents clinically as diabetes without dyslipidemia, as reflected by the index patient. In contrast, HNF1A-MODY was aggravated in the brother by classical insulin resistant type 2 diabetes or features of metabolic syndrome indicated by dyslipidemia with severe hypertriglyceridemia and hepatic steatosis.

Family history and triglyceride concentrations are of special importance in the differentiation of insulin-resistant type 2 diabetes and HNF1A-MODY. In general, type 2 diabetes is characterized by significantly higher triglyceride values and lower high-density lipoprotein cholesterol compared with HNF1A-MODY or non-diabetics, with a high-density lipoprotein cholesterol > 1.12 mmol/l discriminates HNF1A-MODY from type 2 diabetes with 75 % sensitivity and 64 % specificity [17].

On the other hand increased circulating serum lipids are also a combinatory risk factor to various metabolic diseases such as type 2 diabetes. To investigate whether circulating lipids are of special importance in such combined diabetes types, we challenged the affected siblings with lipid and glucose tolerance tests, but gained no further insight beside the present basal clinical features of type 2 diabetes or a more severe insulin-resistant phenotype in the affected brother. Therefore these investigations did not shed light on further therapeutic approaches except therapy for type 2 diabetes.

In our patients, one possible hypothesis for the differences in lipid metabolism observed might be the differing distribution of the maternal genetic predisposition for type 2 diabetes (e.g. in heterozygous states). In combination with the genetic predisposition of the HNF1A-MODY-relevant mutations, this resulted in an exponential metabolic risk, which may be triggered by lifestyle factors. Although in cases with occurrence of type 2 diabetes and HNF1A-MODY-relevant mutations such as this, treatment of type 2 diabetes is the major clinical aim, therapy should be optimized according to the patients’ genetic profile because a MODY-relevant mutation alters the therapeutic needs. Mutations affecting *HNF-1α* transcriptional activity interfere with activation of cellular *HNF-1α* targets, which has been shown to decrease the uptake of sulfonylurea, resulting in increased circulating levels [18]. Therefore patients with HNF1A-MODY have been shown to be hypersensitive to sulfonylurea therapy [19, 20], which makes sulfonylurea the therapy of choice. Nevertheless, tight dose control may be needed for the individual patient. Taken together, the presence of common type 2 diabetes features should not detract from the possibility of MODY in patients with a striking autosomal dominant family history, in order to optimize the individual therapy.

## Consent

Written informed consent was obtained from the patient for publication of this Case report and any accompanying images. A copy of the written consent is available for review by the Editor of this journal.
